# An Accurate and Efficient Time Delay Estimation Method of Ultra-High Frequency Signals for Partial Discharge Localization in Substations

**DOI:** 10.3390/s18103439

**Published:** 2018-10-13

**Authors:** Pengfei Li, Kejie Dai, Tong Zhang, Yantao Jin, Yushun Liu, Yuan Liao

**Affiliations:** 1 School of Electrical and Mechanical Engineering, Pingdingshan University, Pingdingshan 467000, China; pengfei9966@126.com (P.L.); zhangtong_pds@163.com (T.Z.); 15637510195@163.com (Y.J.); 2 Anhui Grid Co., Anhui Electric Power Research Institute, Hefei 230022, China; silencelys@163.com; 3 Department of Electrical and Computer Engineering, University of Kentucky, Lexington, KY 40506, USA; yuan.liao@uky.edu

**Keywords:** PD localization, efficiency and accuracy of TD estimation, full-wavefront extraction method, improved cross-correlation algorithm

## Abstract

Partial discharge (PD) localization in substations based on the ultra-high frequency (UHF) method can be used to efficiently assess insulation conditions. Localization accuracy is affected by the accuracy of the time delay (TD) estimation, which is critical for PD localization in substations. A review of existing TD estimation methods indicates that there is a need to develop methods that are both accurate and computationally efficient. In this paper, a novel TD estimation method is proposed to improve both accuracy and efficiency. The TD is calculated using an improved cross-correlation algorithm based on full-wavefronts of array UHF signals, which are extracted using the minimum cumulative energy method and zero-crossing points searching methods. The cross-correlation algorithm effectively suppresses the TD error caused by differences between full-wavefronts. To verify the method, a simulated PD source test in a laboratory and a field test in a 220 kV substation were carried out. The results show that the proposed method is accurate even in case of low signal-to-noise ratio, but with greatly improved computational efficiency.

## 1. Introduction

The early faults of high-voltage equipment in substations can be detected effectively by locating the partial discharge (PD) source based on the ultra-high frequency (UHF) method [1]. The time difference of arrival (TDOA) method is commonly used as the localization algorithm [2]. The key of the TDOA method is to calculate the time delay (TD) between any two UHF signals captured by the sensor array. With three or four TDs and the coordinates of the sensor array, the localization equations can be established, from which the coordinate of the PD source can be obtained. The accuracy of the TDs has a direct impact on positioning accuracy [3]. Improving TD accuracy has received substantial attention in the field of PD localization in substations.

Conventional TD estimation methods include the threshold method [4], cumulative energy method [5], TD estimation method based on the Akaike information criterion (AIC) [6], and the cross-correlation algorithm [7]. The first three methods have a common feature, that is, the onset time is extracted from the UHF signal with respective methods, then TD is obtained by calculating the difference between the two onset times. The principle of the cross-correlation method is the construction of a cross-correlation function of the two UHF signals, and the offset of the maximum of the function is the TD of the two signals [8]. Theoretically, the TD can be accurately and efficiently calculated using these methods. However, in real substations, errors in TD estimation may occur due to two factors. First, the signal-to-noise ratio (SNR) of the UHF signal is reduced because of periodic narrowband noise and Gaussian white noise, and the onset time is changed with the SNR [9]. Therefore, the accuracy of the first three methods must be affected. Second, the consistency of the UHF array signals is severely reduced by the multipath effect [10,11], which affects the accuracy of the cross-correlation algorithm [12]; nevertheless, this algorithm has certain advantages when the SNR is low.

To obtain an accurate TD, many improved TD estimation methods have been developed. In [13], an improved threshold method was proposed, in which the high energy component of UHF signals was extracted using wavelet decomposition method, and the threshold value was set based on the wavelet coefficient. Subsequently, the onset time of the UHF signal could be obtained. However, the high energy component of the captured UHF signal is dominated by the field noise when the SNR is low, and hence the onset time cannot be obtained with reasonable accuracy. In [14], the continuous wavelet transform (CWT)-based binary map method was proposed, in which the onset time is determined by the earliest non-zero pixel of the binary map obtained by Otsu’s method based on the CWT. The problem of the method is same to the threshold method, and the earliest non-zero pixel is affected by noise. In [6], part of the UHF signal was extracted using a time-domain window, which consists of the wavefront, and subsequently, TD is calculated using the cross-correlation algorithm based on the two extracted signals. When the effective wavefronts in the two parts are different, TD estimation accuracy is inevitably affected. In [15], a TD estimation method based on the interpolation cross-correlation algorithm was proposed to reduce the error generated by limiting the system sample rate, which enhanced the accuracy of TD estimation to a certain degree. However, the error caused by inconsistency in the array UHF signals has not been effectively solved. In [16], a TD estimation method using the cross-correlation algorithm based on a half-wavefront was proposed, which was used to locate the PD source in air-insulated substations. Two problems may affect this method: first, the half-wavefront extraction approach is complex, and this step takes most of the TD estimation time. Second, the half-wavefront extraction result is affected by the SNR. To resolve the influence of non-Gaussian noise on the cross-correlation algorithm, a method based on high-order statistics was proposed in [12]. This method effectively mitigates the effect of field noise. The UHF signal is divided into several segments, whose higher-order cumulants is calculated. The computational burden is large, which severely reduces efficiency. Designing a method that is both accurate and computationally efficient is desirable and challenging.

To improve the accuracy and efficiency of TD estimation and to develop a method applicable to realistic substation conditions, a novel TD estimation method is proposed in this paper. First, the initial time range of the effective UHF signal is obtained using the minimum cumulative energy method. Second, the full-wavefront of the UHF signal is extracted using the proposed zero-crossing point searching method. Third, TD is calculated using an improved cross-correlation algorithm. To verify the method, a simulated PD source was established in the laboratory, and array UHF signals with different SNRs were used as the samples for testing the method. A field test in a 220 kV substation with a real PD source was subsequently carried out. The accuracy and efficiency of the method were verified by comparing it with selected published methods.

## 2. TD Estimation Method by Using Improved Cross-Correlation Algorithm Based on Full-Wavefronts of UHF Signals

### 2.1. Characteristics Analysis of Array UHF Signals

#### 2.1.1. Model of Array UHF Signals

To locate the PD source in the substation, a UHF sensor array was used to capture the signal emitted by the PD source. UHF signals typically comprise three components: the PD electromagnetic signal, periodic narrowband noise and the white noise, as shown in Equation (1). The *y_i_*(*t*) is the UHF signal received by the *i*-th sensor, *v_i_*(*t*) is the electromagnetic signal radiated from the PD source, *g_i_*(*t*) is the periodic narrowband noise, and *w_i_*(*t*) is the white noise. The frequency of UHF signals is within the range of 300 MHz–3 GHz [17]. Considering the frequencies of wireless communication signals used in China, the frequencies of the periodic narrowband noise are 470 MHz, 900 MHz and 1800 MHz [18]. White noise is generated by the heating effect of high-voltage apparatus:

(1) $$y_{i}(t)=v_{i}(t)+g_{i}(t)+w_{i}(t)\;$$

**Figure 1:**
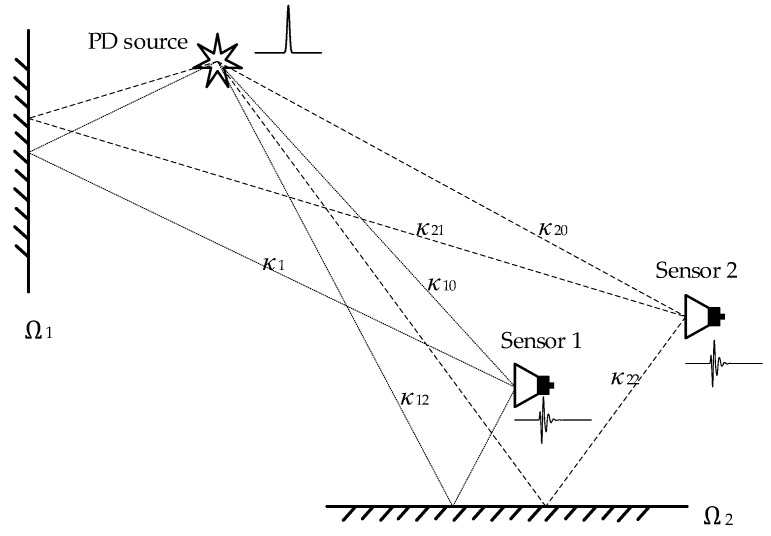
The diagram of multipath propagation of UHF signal.

Because of the substation’s complex environment, the propagation paths of UHF signals received by different sensors differ, as shown in Figure 1, where Ω represents the reflection surface, *κ_xx_* represents different propagation paths, and *v_i_*(*t*) is the superposition of the direct wave and several reflected waves. *v_i_*(*t*) can be expressed as Equation (2), where *e_i_*_0_(*t*) is the direct wave; *θ* and *φ* are the azimuth angle and pitch angle, respectively, between the sensor and any point in the space; and *h*(*θ*_0_, *φ*_0_, *t*) is the transfer function of the sensor at bearing (*θ*_0_, *φ*_0_). * is the convolution operator, *ζ_k_* is the attenuation coefficient of the electromagnetic signal, *e*(*t* + Δ*t_κ_*) is the expression of the reflected wave in the time domain, Δ*t_κ_* is the TD between the direct wave and reflected wave, and *h*(*θ_κ_*, *φ_κ_*, *t*) is the transfer function of the sensor at bearing (*θ_κ_*, *φ_κ_*).

(2) $$v_{i}(t)=e_{i0}(t)*h(\theta_{0},\varphi_{0},t)+\sum_{\kappa =1}^{n}\zeta_{\kappa }e_{i}(t+\Delta t_{\kappa })*h(\theta_{\kappa },\varphi_{\kappa },t)\;$$

The positions of the sensors in the sensor array differ, thus, the propagation paths of the UHF signals received by different sensors differ, and the bearings (*θ*, *φ*) of each signal differ, which would lead the inconsistencies of the array UHF signals. According to Figure 1, the propagation path of the reflected wave is longer than that of the direct wave, so the reflect wave is at the end of the UHF signal. According to Equation (2), if the reflection surfaces are different, the bearings (*θ_κ_*, *φ_κ_*) will be different. The gain of the sensor is related to directionality [19]. Therefore, the inconsistencies of the array UHF signals caused by the multipath effect exist at the end of the signals, and the wavefronts are unaffected or only slightly affected.

#### 2.1.2. Measured Array UHF Signals

**Figure 2:**
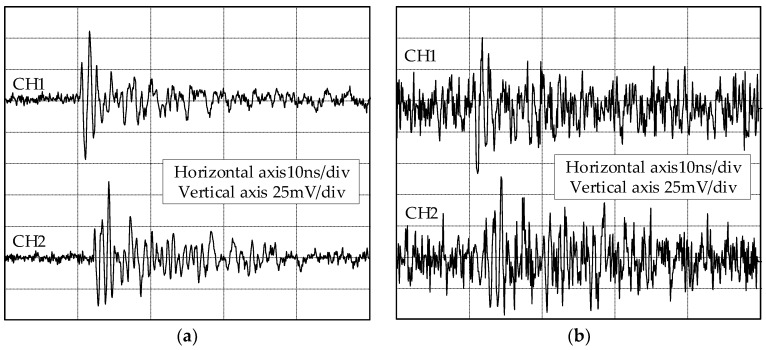
Array UHF signals radiated from the PD defect model and the noisy array UHF signals: (**a**) array UHF signals; (**b**) noisy array UHF signals.

Figure 2 depicts the array UHF signals radiated from a PD defect model in the laboratory. The signals were captured using two Vivaldi antennas [20] and were recorded by a LeCry WR640Zi oscilloscope with a bandwidth of 4 GHz and a sampling rate of 20 GS/s. The distance between the two antennas was 1.5 m, and the distances between the PD defect model and the two antennas were 4.24 and 3.71 m, respectively. The propagation speed of the electromagnetic wave was 3.00 × 10^8^ m/s, and thus the TD of the two UHF signals was 1.767 ns, or 35–36 samples. To verify the effectiveness of the TD estimation method at a low SNR, the field noise was measured at the substation, and the noise was added to the array UHF signals. The noisy UHF signals are presented in Figure 2b, and the SNR was 3.86 dB.

### 2.2. TD Estimation Method Using the Improved Cross-Correlation Algorithm Based on the Full-Wavefront

According to the analysis in Section 2.1.1, the wavefront of the UHF signal has the least distortion; furthermore, it contains the largest proportion of the direct wave energy. If the full-wavefront is used for TD estimation instead of the complete UHF signal, then the error caused by the multipath effect can be minimized. The cross-correlation algorithm can reduce the influence of uncorrelated noise, leading to more accurate TD estimate. However, the effect of signal consistency on the result must be considered. The methods for accurately and effectively extracting the wavefront from the noisy UHF signal and improving the cross-correlation algorithm are key to the TD estimation method.

#### 2.2.1. Full-Wavefront Extraction Method

To obtain an accurate full-wavefront, periodic narrowband noise and white noise should be considered. The full-wavefront extraction procedure is as follows:

*Step 1*: Suppressing the white noise *w*(*t*). The original UHF signal *y_i_*(*n*) is filtered using a neighborhood smoothing filter, and the signal is recorded as *y_i_*′(*n*). The neighborhood smoothing filter is defined in Equation (3), where *M* is the width of the neighborhood:

(3) $$y_{i}^{\prime }(n+\frac{M}{2})=\frac{1}{M}\sum_{m=1}^{M}y_{i}(n+m)\;$$

*Step 2*: Suppressing the periodic narrowband noise *g*(*t*).

**Figure 3:**
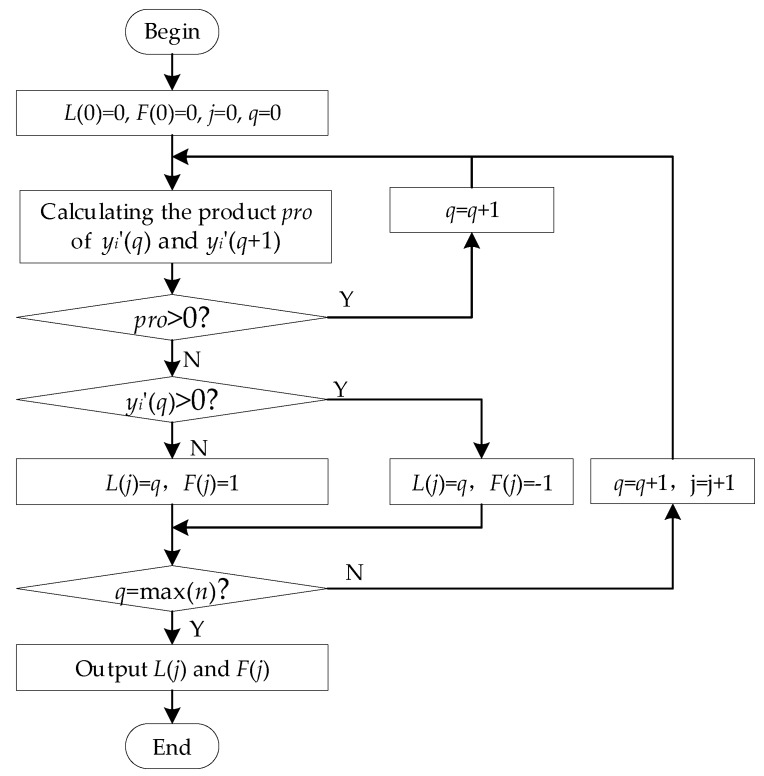
Flow chart of the zero-crossing point searching method.

*Step 2.1*: The cumulative energy curve of *y_i_*′(*n*) is calculated using Equation (4); subsequently, the minimum cumulative energy curve of *y_i_*′(*n*) is calculated using Equation (5), where *E_N_* is the maximum of *E*(*n*). The sampling number of the minimum of *E_min_*(*n*) is the apparent onset time of *y_i_*(*n*); the sampling number is subsequently extracted and recorded as *N_min_*. Note that the apparent onset time is not the actual onset time, which is affected by the noise and the procedure in Step 1.
*Step 2.2*: The noise frame of the UHF signal *w*′(*n*) is extracted from the first sampling point to Nmin. The zero-crossing points and crossing direction are obtained using the method shown in Figure 3, where *L*(*j*) is the sampling number of the zero-crossing point, *F*(*j*) is the crossing direction of the zero-crossing point, *F*(*j*) = 1 indicates the rising edge, and *F*(*j*) = −1 indicates the falling edge.
  (4) $$E(n)=\sum_{p=0}^{n}y\prime^{2}(p)\;$$
  (5) $$E_{\min}(n)=E(n)-n\frac{E_{N}}{N}=\sum_{m=0}^{n}\left[y\prime^{2}(m)-n\frac{E_{N}}{N}\right]\;$$
*Step 2.3*: Depending on the results acquired in Step 2.2, the signal *w*′′(*n*) is extracted from the noise frame *w*′(*n*) between the first zero-crossing point *L*(0) and the last zero-crossing point, which has the same crossing direction as *L*(0). The signal *w*′′(*n*) is extended *r* times, and the length of the extended signal is checked to ensure it is equal to the length of *y_i_*(*n*) from point *L*(0) to the end of the signal, which is recorded as *w*′′′(*n*). The signal from *y_i_*′(*n*) between *L*(0) and the end is extracted; subsequently, the difference between the extracted signal and *w*′′′(*n*) is calculated and recorded as *y_i-dif_*′(*n*). Subsequently, *L*(0) zeros are padded at the front of *y_i-dif_*′(*n*), which is recorded as *y_i_*′′(*n*), and the signal *y_i_*′′(*n*) is the UHF signal after the periodic narrowband noise is suppressed.

*Step 3*: Extracting the full-wavefront

*Step 3.1*: The minimum cumulative energy curve of *y_i_*′′(*n*) is calculated using Equations (4) and (5), and the sampling number *N*′′*_min_* is extracted, which corresponds to the minimum of the curve. Two signals (duration: 3 ns) are extracted before and after *N*′′*_min_*, and the extracted signal is the effective signal frame, which is recorded as *y_iwf_*′′(*n*).
*Step 3.2*: The signal segment of *y_i_*′′(*n*) is extracted from the start of *y_i_*′′(*n*) to the sample *N*′′*_min_*, and the mean value of the absolute value of all crests and troughs is calculated, which is recorded as *A*_ave_. The zero-crossing points of *y_i_*′′(*n*) are obtained using the method presented in Figure 3, and the absolute value of the crest and trough between every two zero-crossing points is counted. With *δ*·*A*_ave_ as the threshold, to find the first absolute value that is larger than *δ*·*A*_ave_, the sampling number of the previous zero-crossing point before the absolute value is the start of the full-wavefront. The third zero-crossing point is the end of the full-wavefront. With the start and the end samples, the full-wavefront is extracted from the original signal *y*(*n*). This indicates that the full-wavefront contains a crest and a trough.

**Figure 4:**
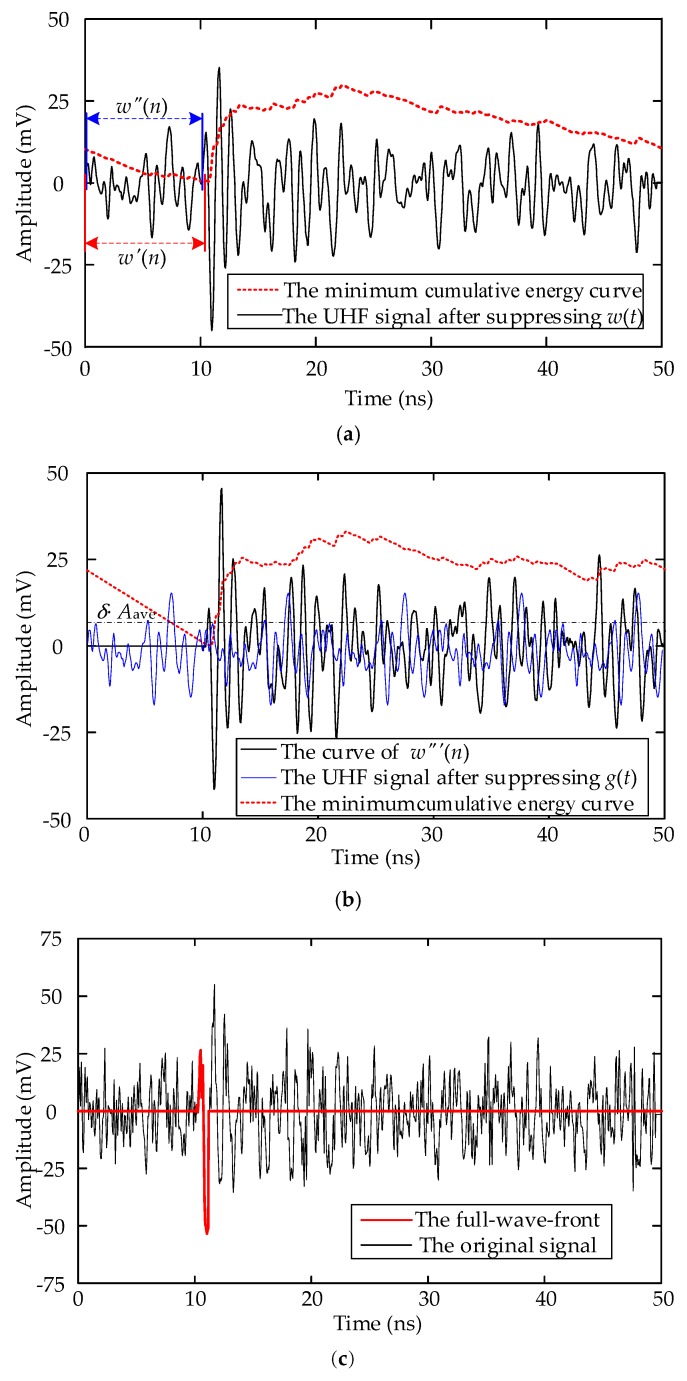
Schematic of the full-wavefront extraction procedure: (**a**) Results of Step 1; (**b**) Results of Step 2; (**c**) Results of Step 3.

Taking the first signal in Figure 2b as an example, the full-wavefront extraction procedure is illustrated in Figure 4. 

Figure 4a presents the result of suppressing the white noise, and the neighborhood smoothing filter parameter *M* is set as 5. Figure 4b presents the result of suppressing the periodic narrowband noise and the full-wavefront extraction process. In Step 2, the value of *δ* is set as 1.1. Figure 4c shows the extracted full-wavefront.

#### 2.2.2. TD Calculation Using the Improved Cross-Correlation Algorithm

**Figure 5:**
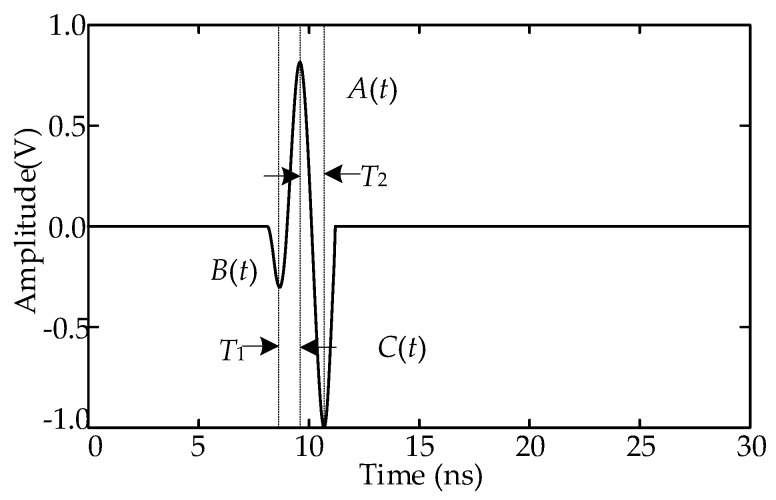
Analysis of the full-wavefront.

As explained in Section 2.1, the values of the wavefronts of the array UHF signals differ because of the difference in the bearings and directionality of the UHF sensor. During full-wavefront extraction, only the parameter *δ* needs to be set manually. Because of the difference among the array UHF signals, if the parameter *δ* is unreasonable, then the array full-wavefronts will deviate. Assume that the value of *δ*·*A*_ave_ is greater than the amplitude of the first half wavefront; consequently, the full-wavefront extraction result will deviate, as described in Figure 5. The wave in Figure 5 can be divided into three half-waves: *A*(*t*), *B*(*t*), and *C*(*t*). The corect full-wavefront is *A*(*t*) + *B*(*t*), and the full-wavefront with deviation is *A*(*t*) + *C*(*t*). Only if the extracted full-wavefronts are all correct or have the same deviations can the correct TD be obtained using the cross-correlation algorithm. However, the parameter *δ*·*A*_ave_ cannot satisfy all the UHF signals.

To obtain the correct TD when the two wavefronts are inconsistent, the cross-correlation algorithm should be improved. The half wavefront is recorded as *v_wf_*(*t*); assume that the center times of *A*(*t*), *B*(*t*), and *C*(*t*) are *t*, *t* − *T*_1_, and *t* + *T*_2_, respectively. The expressions of the three half-waves are presented in Equation (6), where *a*, *b*, and *c* are the amplitudes of *A*(*t*), *B*(*t*), and *C*(*t*), respectively.

(6) $$\left\{ \begin{array}{l} A(t)=a\cdot v_{wf}(t)\\ B(t)=b\cdot v_{wf}(t-T_{1})\\ C(t)=c\cdot v_{wf}(t+T_{2}) \end{array}\right.\;$$

In the case of deviation, the expression of the two full-wavefronts *s*_1_(*t*) and *s*_2_(*t*) is as Equation (7), where *ζ* is the ratio of the amplitudes of the two wavefronts:

(7) $$\left\{ \begin{array}{l} s_{1}(t)=A(t)+B(t)=a\cdot v_{wf}(t)+b\cdot v_{wf}(t-T_{1})\\ s_{2}(t)=\zeta \cdot A(t+\tau )+\zeta \cdot C(t+\tau )=\zeta a\cdot v_{wf}(t+\tau )+\zeta c\cdot v_{wf}(t+T_{2}+\tau ) \end{array}\right.\;$$

The cross-correlation function of *s*_1_(*t*) and *s*_2_(*t*) is shown as Equation (8), where *E* is the expected operator:

(8) $$\begin{array}{ll} R_{12}(\tau ) & =E(s_{1}(t)\cdot s_{2}(t))\\ & =E((a\cdot v_{wf}(t)+b\cdot v_{wf}(t-T_{1}))\cdot (\zeta \cdot a\cdot v_{wf}(t+\tau )+\zeta \cdot c\cdot v_{wf}(t+T_{2}+\tau )))\\ & =\zeta \cdot (a^{2}\cdot E(v_{wf}(t)\cdot v_{wf}(t+\tau ))+ac\cdot E(v_{wf}(t)\cdot v_{wf}(t+T_{2}+\tau ))+\\ & \;ab\cdot E(v_{wf}(t+\tau )\cdot v_{wf}(t-T_{1}))+bc\cdot E(v_{wf}(t-T_{1})\cdot v_{wf}(t+T_{2}+\tau ))) \end{array}$$

The result of Equation (8) is the sum of the cross-correlation functions, *A*(*t*) and *A*(*t + τ*), *A*(*t*) and *C*(*t + τ*), *A*(*t + τ*) and *B*(*t*), and *B*(*t*) and *C*(*t + τ*). According to the principle of the cross-correlation algorithm, when *t* = −*τ*, the cross-correlation function of *A*(*t*) and *A*(*t + τ*) reaches the extremum value of *ζ*·*a*^2^, which is the correct TD of *s*_1_(*t*) and *s*_2_(*t*). The time and values of the extremum of the four cross-correlation functions are listed in Table 1. The data in the table indicate that *ζ*·*a*^2^ and *ζ*·*bc* are both positive numbers, and that *ζ*·*ab* and *ζ*·*ac* are both negative numbers. Thus, theoretically, there are two crests and two troughs on the cross-correlation curve. Because the values of *T*_1_ and *T*_2_ are close, the two troughs overlap. The amplitudes of the two crests are related to *ζ*·*a*^2^ and *ζ*·*bc*, respectively. The correct TD corresponds to the crest whose amplitude is *ζ*·*a*^2^, and the correct TD can be obtained by judging the magnitude of *ζ*·*a*^2^ and *ζ*·*bc*.

How to determine parameter *a* is key to the improved cross-correlation algorithm. Per the results of Step 3.1 in Section 2.2.1, the sampling number *N*′′*_min_* is close to the actual onset time of the UHF signal. The difference between *N*′′*_min_* and the sample point of the first extremum was calculated, and the wavefront with a smaller difference was the correct one to be used for determining parameter *a*.

**Figure 6:**
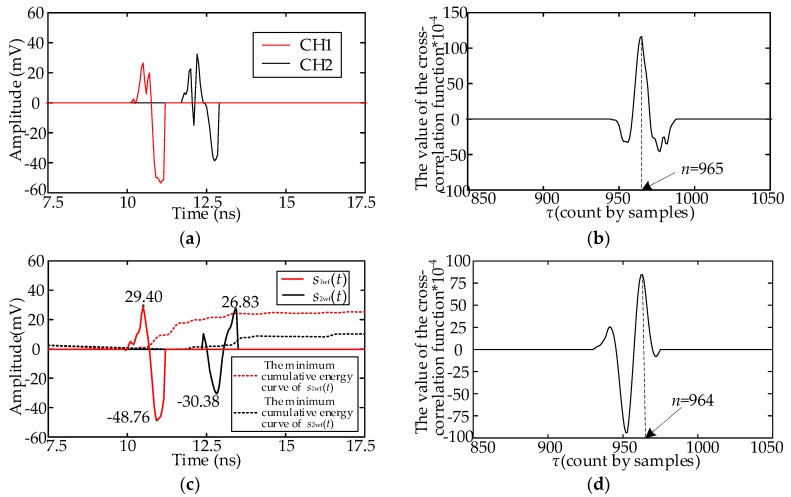
Improved cross-correlation algorithm; (**a**) Two full-wavefronts of the signals in Figure 2b; (**b**) Cross-correlation function curve of the signal in (**a**); (**c**) Two full-wavefronts of another set of UHF signals; (**d**) Cross-correlation function curve of the signal in (**c**).

Consider the signals in Figure 2b as an example. The full-wavefront and cross-correlation curve are presented in Figure 6a,b, respectively. The peak of the cross-correlation curve occurs at sample point 965. The numbers of samples for the two UHF signals are both 1000. The estimated number of samples for the TD result is 35, which is the correct value. The full-wavefronts of another two UHF signals with deviation and minimum cumulative energy curve are listed in Figure 6c, and the full-wavefronts are recorded as *s*_1wf_(*t*) and *s*_2wf_(*t*). The cross-correlation curve is illustrated in Figure 6d. According to the aforementioned estimation method, *s*_1wf_(*t*) is the correct full-wavefront. The amplitudes of the crests and troughs of the two full-wavefronts are determined: max(*s*_1wf_(*t*)) = 29.40 mV, min(*s*_1wf_(*t*)) = −48.76 mV, max(*s*_2wf_(*t*)) = 26.83 mV, and min(*s*_1wf_(*t*)) = −30.38 mV. Three extremums are shown on the curve. This indicates that *a*^2^ = min(*s*_1wf_(*t*)) · min(*s*_1wf_(*t*)) and *bc* = max(*s*_1wf_(*t*)) · max(*s*_2wf_(*t*)). Because *a*^2^ > *bc*, the sampling point of the second peak is selected, which is 964. The TD estimation result is 36, which is consistent with the real TD.

## 3. Comparative Analysis of Several TD Estimation Methods

**Figure 7:**
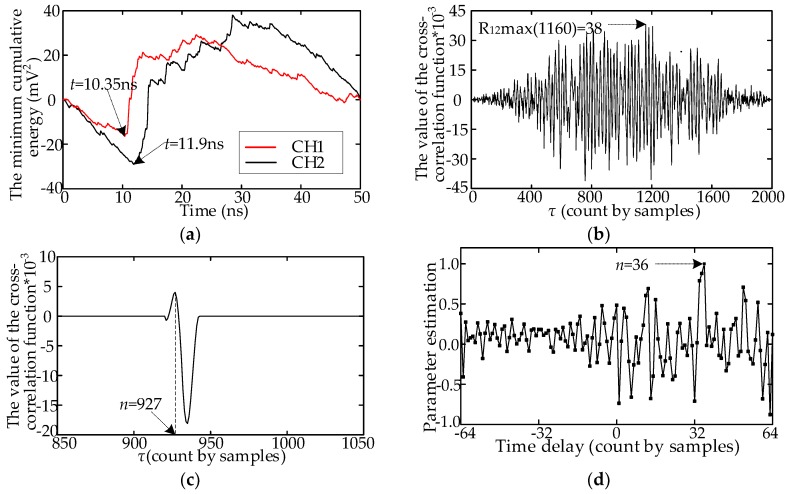
TD estimation results of the four published methods; (**a**) Method A; (**b**) Method B; (**c**) Method C; (**d**) Method D.

To verify the accuracy and efficiency of the proposed method in this paper, the performance of the proposed method was compared with that of four published methods: the minimum cumulative energy method [5], cross-correlation algorithm, cross-correlation algorithm based on half-wavefront [16], and TD estimation method based on high-order statistics [12]. The four methods are described in Table 2. With the array UHF signals in Figure 2b as an example, the TD estimation results provided by the four methods are listed in Figure 7. In Figure 7a, the TD is 1.55 ns, calculated using Method A, and the error is 5 samples. In Figure 7b, the number of samples at the peak of the cross-correlation function curve is 1160. The TD is −160 samples, and the error is because of the inadequate consistency of the two UHF signals. The TD calculated using Method C is 73 sampling points, as shown in Figure 7c, and the error is 38 samples, which is because of the deviation between the two half-wavefronts. In Figure 7d, the TD calculated using Method D is 36, which is consistent with the actual TD.

Fifty array UHF signals were measured in the laboratory, and field noise of various amplitudes was added. These signals were then used to verify the accuracy and efficiency of the five methods. The SNRs were set to 25, 20, 15, 10 and 5 dB. The means and standard deviations of the 50 TD estimation results were used to evaluate accuracy, and the total computational time was used to evaluate efficiency. The computer configuration was as follows: an Intel Core i5-4460 system with 3.2 GHz of clock speed, 8 GB of RAM, and a 64-bit Windows 7 operating system. The means, standard deviations, and time consumptions for the 50 array UHF signals are listed in Table 3.

As the SNR decreased, the TD estimation accuracy of the five methods also decreased. For SNR = 25 dB, the mean value derived using Method B deviated substantially from the actual TD, whereas other methods could obtain accurate results. This indicates that the array signal consistency has stronger effect on the cross-correlation algorithm. When the SNR ≤ 10 dB, the standard deviations of Methods A and C increased sharply, indicating that field noise has a greater effect on these two methods than on other methods. For Method A, the position of the trough in the minimum energy accumulation curve changed depending on field noise. For Method C, the amplitude of the noise exceeded the amplitude of the half-wavefront, which caused the deviation of the half-wavefront extraction. In this case, the conventional cross-correlation algorithm will fail to obtain the correct time delay. The proposed method in this paper and Method D are more accurate than the other three methods. This indicates that the two methods suppress noise and array signal consistency effectively.

The time consumptions of the proposed method and methods A, B, and C have the same order of magnitude, and the time consumptions are all less than 200 ms. The time consumption of Method C is greater than that of the proposed method because the time-frequency analysis is used to extract the half-wavefront, which is a time-intensive process. The time required for Method D is 3860.5 s, which is 40,213 times as much as that for the proposed method. Because numerous matrix operations are performed during the TD calculation, the efficiency of the algorithm is severely reduced. By contrast, only the basic array operations are used in the proposed method, thus greatly improving the computational efficiency. In summary, the proposed TD estimation method is highly accurate and efficient for array UHF signals with a low SNR.

## 4. Field Test in a Substation

### 4.1. Test Arrangement

**Figure 8:**
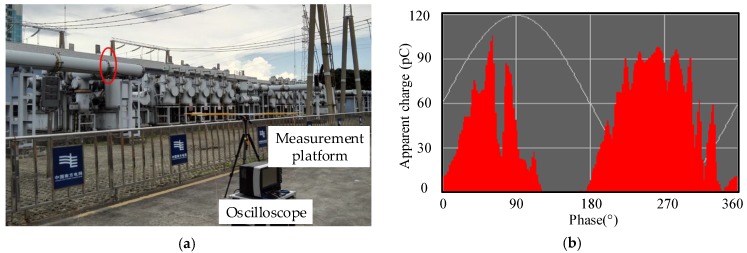

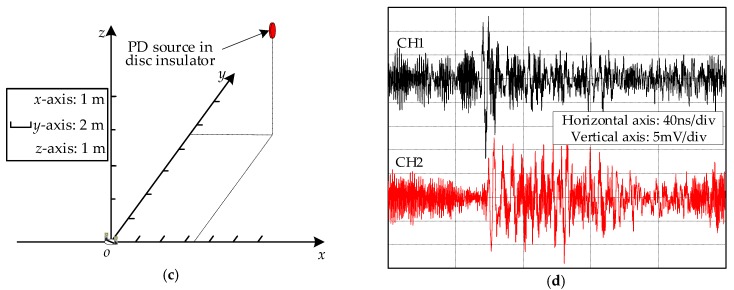
Field test site and measurement result of the disc insulator PD: (**a**) Field test site and test arrangement; (**b**) Online monitoring result; (**c**) Coordinate system of the field test site arrangement; (**d**) UHF signals captured by the antenna array.

To verify the effectiveness of proposed method, a field test in a 220 kV substation was carried out. The field test setup is shown in Figure 8a. The measurement system comprised an antenna array, a rotating platform, and a data acquisition system. The parameters of the antenna array and oscilloscope were identical to those described in Section 2.1.2. The antenna array was placed on the rotating platform, whose center coincides with that of the antenna array. A PD source was placed in a disc insulator with an air-gap defect, as marked by the red ellipse in Figure 8a. The result of the online monitoring system is presented in Figure 8b. 

In the substation, the coordinate system was established, and the plane where the array sensors are placed is set as the *xoy* plane. The location range of the disc insulator was {*x*|*x* ∈ (3.3, 3.4)}, {*y*|*y* ∈ (8.55, 9)}, and {*z*|*z* ∈ (2.6, 3)} in meters. The origin point was selected as the measurement point, *o* (0, 0, 0). One of the measured array UHF signals is listed in Figure 8d, and the rotation angle of the antenna array is −15°. The TD range of the two UHF signals is 0.5–0.6 ns, and the corresponding samples are theoretically 10–12. The amplitude of the field noise is close to that of the signal, and the SNR is low.

### 4.2. Result Analysis of the TD Estimation

**Figure 9:**
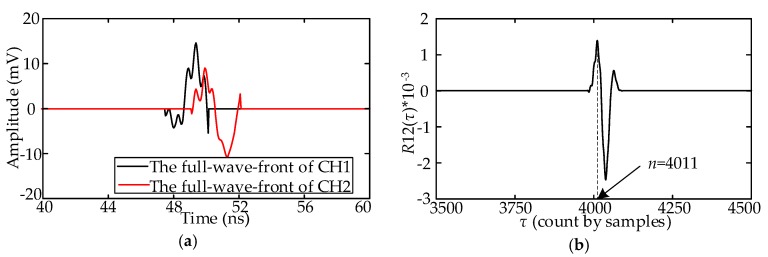
TD estimation result of the field UHF signal using the proposed method: (**a**) Extracted full-wavefronts; (**b**) Cross-correlation function curve.

**Figure 10:**
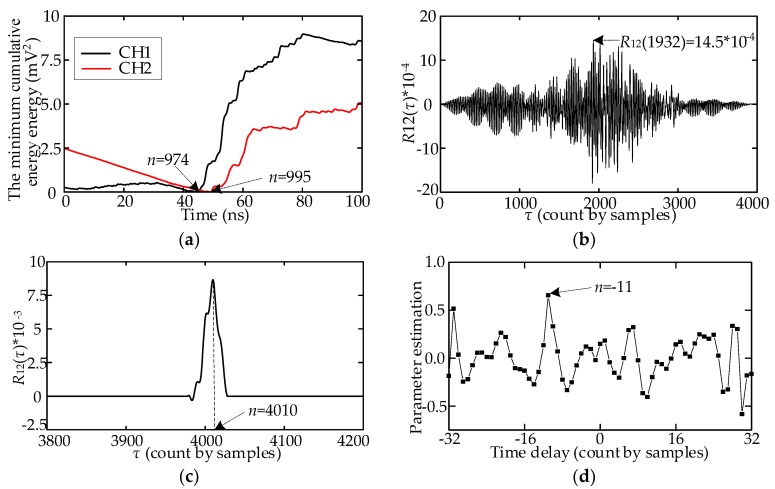
TD estimation results of the four methods: (**a**) Method A; (**b**) Method B; (**c**) Method C; (**d**) Method D.

The TD is calculated as follows: First, the full-wavefronts are extracted, as shown in Figure 9a, and a deviation separates the two full wavefronts. Figure 9b illustrates the cross-correlation function curve. According to Section 2.2.2, the sampling point of the maximum is 4011; thus, the TD is 11 sampling points (0.55 ns). Figure 10 presents a comparison of the TD estimation result of the four published methods. The calculated TD was 1.05, 3.4, 0.5, and 0.55 ns. The results calculated using Methods C and D are accurate. At this rotating angle, TD estimation results obtained considering 100 array UHF signals as samples are shown in Table 4. Method B has the largest error. The SNR of some UHF signals is excessively low, which leads to the deviation in the inflection point and the half-wavefront. Thus, the means of TDs calculated using Methods A and B deviate from the actual TD. 

The TD obtained using Method C is close to the actual result, while the standard deviation is large. This indicates that some of the calculation results have large deviations. Both Method D and the proposed method have obtained TD with higher accuracy. In terms of computational efficiency, time consumption is 41,270 times as much as that of the proposed method.

## 5. Conclusions

In this paper, a TD estimation method for locating PD sources in substations is proposed, in which the full-wavefront of the UHF signal is extracted to replace the original UHF signal. The cross-correlation algorithm was improved to enhance TD accuracy. A simulated model was established, and a field test was conducted to verify the method. The conclusions are summarized as follows:

(1) The full-wavefront was extracted for calculating the TD, which reduces the influence of the field noise and the inconsistency of the array UHF signals and thus effectively improves the accuracy of TD estimation.
(2) TD estimation efficiency was enhanced using the proposed full-wavefront extraction method and improved cross-correlation algorithm, making it more suitable for field applications.
(3) An experimental platform with a PD defect model was established in the laboratory. Three hundred array UHF signals with different SNRs were used as the samples to verify the proposed method, and the results of the proposed method were compared with that of four published methods. The proposed method yielded accurate TD estimation based on high-order statistics. Regarding efficiency, computational time was reduced from 3,860,500 ms to 96 ms, and the efficiency increased more than 40,000 times.
(4) A field test was carried in a 220 kV substation. An air-gap discharge in a disc insulator was used as the testing target. Compared with the four previously published methods, the proposed method yielded the most accurate TD with lowest time requirement, indicating the high accuracy and efficiency of the proposed method.

## Figures and Tables

**Table 1:** Time and values of the extrema of four cross-correlation functions.

Cross-Correlation Function	Time of the Extremum	Value of the Extremum
A(*t*), A(*t* + *τ*)	−*τ*	*ζ*·*a*^2^
A(*t*), C(*t + τ*)	−*T*_1_ − *τ*	ζ·ac
A(*t* + *τ*), B(*t*)	−*T*_2_ − *τ*	*ζ*·*ab*
B(*t*), C(*t* + *τ*)	−*T*_1_ − *T*_2_ − *τ*	*ζ*·*bc*

**Table 2:** Descriptions of the four published TD estimation methods.

TD Estimation Method	Method Name	Principle
**Method A**	Minimum cumulative energy method	(1) The minimum cumulative energy curve is calculated using Equations (4) and (5). (2) The sampling number correspond to the minimum of the curve is determined, which is the start of the UHF signal frame. (3) The difference in the sampling numbers of the two signals is calculated, which is the TD.
**Method B**	Cross-correlation algorithm	(1) The cross-correlation function of two signals is calculated. (2) The offset of the maximum value of the cross-correlation function is determined, which is the TD.
**Method C**	TD estimation method proposed in [16]	(1) The time-frequency transform of the UHF signal is carried out. (2) The signal in the range 0.5–0.8 GHz is extracted, and the signal is reconstructed. (3) The signal containing the wavefront is extracted on the basis of the reconstructed signal, and the number and positions of the crest and trough are found. (4) The half-wavefront is extracted, and the TD is calculated using the cross-correlation algorithm.
**Method D**	TD estimation method based on high-order statistics	The principle of this method is described in [12].

**Table 3:** TD estimation results for the five methods.

TD Estimation Method		The Type of the UHF Signals						Time Consumed (ms)
		Original Signal	25 dB	20 dB	15 dB	10 dB	5 dB	
**Method A**	Mean	35.38	35.52	35.52	35.54	35.7	35.68	32
	Standard deviation	0.8	0.78	0.75	0.88	1.63	3.05	
**Method B**	Mean	38	38.38	40.34	42.5	42.78	32.68	18
	Standard deviation	8.12	8.32	9.1	9.65	9.64	51.37	
**Method C**	Mean	35.37	35.42	34.98	35.78	40.15	42.68	166
	Standard deviation	0.56	0.63	0.72	0.87	5.58	8.36	
**Method D**	Mean	35.36	35.36	35.36	35.4	35.16	35.28	3,860,500
	Standard deviation	0.59	0.56	0.59	0.6	0.69	0.88	
**The proposed method**	Mean	35.33	35.24	35.24	35.22	35.14	34.98	96
	Standard deviation	0.42	0.39	0.46	0.54	0.66	0.78	

**Table 4:** TD estimation results of the 100 field array UHF signals of the five methods.

TD Estimation Method	Mean of TDs (ns)	Standard Deviation	Time Consumed (ms)
**Method A**	0.6406	0.409	17
**Method B**	1.682	4.478	13
**Method C**	0.517	0.452	213
**Method D**	0.557	0.044	2,352,367
**The proposed method**	0.545	0.042	57

## Abbreviations

The following abbreviations are used in this manuscript:

PD	partial discharge
UHF	ultra-high frequency
TD	time delay
SNR	signal noise ratio
TDOA	time difference of arrival
CWT	continuous wavelet transform
